# *Plxnd1* Expression in Thymocytes Regulates Their Intrathymic Migration While That in Thymic Endothelium Impacts Medullary Topology

**DOI:** 10.3389/fimmu.2013.00392

**Published:** 2013-11-19

**Authors:** Young I. Choi, Jonathan S. Duke-Cohan, Jing Tan, Jingang Gui, Manvendra K. Singh, Jonathan A. Epstein, Ellis L. Reinherz

**Affiliations:** ^1^Laboratory of Immunobiology, Department of Medical Oncology, Dana-Farber Cancer Institute, Boston, MA, USA; ^2^Department of Medicine, Harvard Medical School, Boston, MA, USA; ^3^Department of Hematology, Affiliated Hospital of North Sichuan Medical College, Sichuan, China; ^4^Department of Cell and Developmental Biology, The Cardiovascular Institute, University of Pennsylvania, Philadelphia, PA, USA

**Keywords:** plexind1, thymic development, thymic epithelial cells, angiogenesis, autoimmunity

## Abstract

An important role for plexinD1 in thymic development is inferred from studies of germline *Plxnd1* knockout (KO) mice where mislocalized CD69^+^ thymocytes as well as ectopic thymic subcapsular medullary structures were observed. Given embryonic lethality of the *Plxnd1*^−/−^ genotype, fetal liver transplantation was employed in these prior analyses. Such embryonic hematopoietic reconstitution may have transferred *Plxnd1* KO endothelial and/or epithelial stem cells in addition to *Plxnd1* KO lymphoid progenitors, thereby contributing to that phenotype. Here we use *Plxnd1*^flox/flox^ mice crossed to *pLck*-Cre, *pKeratin14*-Cre, or *pTek*-Cre transgenic animals to create cell-type specific conditional knockout (CKO) lines involving thymocytes (D1ThyCKO), thymic epithelium (D1EpCKO), and thymic endothelium (D1EnCKO), respectively. These CKOs allowed us to directly assess the role of plexinD1 in each lineage. Loss of plexinD1 expression on double positive (DP) thymocytes leads to their aberrant migration and cortical retention after TCR-mediated positive selection. In contrast, ectopic medulla formation is a consequence of loss of plexinD1 expression on endothelial cells, in turn linked to dysregulation of thymic angiogenesis. D1EpCKO thymi manifest neither abnormality. Collectively, our findings underscore the non-redundant roles for plexinD1 on thymocytes and endothelium, including the dynamic nature of medulla formation resulting from crosstalk between these thymic cellular components.

## Introduction

The plexins encode large (∼200 kDa) transmembrane glycoproteins with domain similarity to the scatter factor receptors encoded by the MET gene family and RON receptor tyrosine kinases (1). There are four plexin families (plexin-A, -B, -C, -D) that function as receptors for multiple subfamilies of semaphorins, sometimes in the context of neuropilin-1, and neuropilin-2, to control cell dissociation or attachment, and repulsion or attraction in a variety of tissue types. Approximately 20 vertebrate semaphorins are known, classified in five distinct subfamilies of secreted and surface-bound proteins (1). In the nervous system, the effects of secreted semaphorin-plexin interaction on axonal pathfinding are generally repulsive (1). The cytoplasmic domain of each plexin contains a GTPase activation domain (GAP) of ∼600 aa encompassing a central Rac- or Rho-like binding motif (RBD) (2, 3). These domains have been shown to interact with and regulate the activity of a number of small GTPases including direct binding of Rac1, Rnd1, and RhoD to the RBD and less-well defined sequestration of, or GAP activity against, R-Ras and Rap family members (4–6). The end result is often an effect upon cell motility including an extensive crosstalk with integrin-mediated adhesion (7–9).

In addition to neural development (10), plexinD1 was previously reported to be important in angiogenesis and cardiovascular development (11, 12) with consequent effects upon bone vascularization and skeletogenesis (13, 14). In addition, lymphoid trafficking including migration of thymocytes (15) and movement of B cells into germinal centers (16) is regulated by plexinD1. Specifically, with respect to thymocytes, it is the cortical to medullary trafficking of the positively selected double positive (DP) thymocytes which is disrupted in cells lacking plexinD1 (15). Repopulation of irradiated Ly5 congenic mice with fetal liver-derived hematopoietic stem cells from germline knockout (KO) plexinD1 (*Plxnd1*^−/−^) mice resulted in developing CD69^+^ DP thymocytes that exhibited aberrant trafficking following TCR activation (15). In contrast to DP thymocytes arising from wild-type (WT) transplanted fetal liver progenitors, a large fraction of *Plxnd1*^−/−^ post-selection CD69^+^ DP thymocytes did not migrate toward the medulla as they further differentiate but rather remained in the cortex. Thymi reconstituted with *Plxnd1*^−/−^ hematopoietic progenitors also manifest abnormal ectopic medulla formation, with medullary foci primarily localized to the subcapsular zone.

While recent data showing that the plexinD1 signaling pathway controls both the surface topology of β1 integrin nanoclusters and individual integrin catch bonds readily account for the thymocyte migratory defect (manuscript submitted), the basis of the ectopic medulla formation is unclear. The role of plexinD1 in determining thymic medullary morphogenesis is complicated by its essential regulation of endothelial cell branching during angiogenesis (11, 12, 14), a process of critical importance to thymic structure, impacting entry, and egress of lymphoid cells as well as thymic tissue oxygenation (17–19). Sema3E, a soluble secreted molecule whose primary source within the thymus is the medulla, is the physiological ligand of plexinD1 (15). It is through interaction with plexinD1 that sema3E has been demonstrated to regulate vascular endothelial branching in various tissues (20). Developmental analysis of *Plxnd1* and *Sema3e* germline deletion embryos reveals defects in aorta-pulmonary artery large vessel separation (*truncus arteriosus*) in *Plxnd1*^−/−^ animals but not in the *Sema3e*^−/−^ animals, implying a cardiovascular developmental function for plexinD1 distinct from its sema3E ligand (11). Supporting the complexity of plexinD1 function in angiogenesis, VEGF regulates plexinD1 to limit expression to the front of actively sprouting blood vessels where further signaling may or may not occur dependent upon the relative presence of sema3E (21, 22).

In this report, we analyze the contributions of plexinD1 in differing cellular thymic compartments that collectively foster thymocyte development. Since germline *Plxnd1* deletion is embryonic lethal and thymic reconstitution with stem cells derived from fetal liver may include endothelial progenitors with angiogenic potential as well as other progenitors with epithelial cell potential (23–25), we undertook a systematic analysis of conditional knockout (CKO) mice where cell-specific *Plxnd1* deletion allowed development on an otherwise normal B6 background. This strategy permitted us to delineate the functional spheres of operation of plexinD1 within the thymus. Three different *Plxnd1*^flox/flox^ models were developed where transgene expression of *Cre* recombinase from the *pLck* promoter (26) in one resulted in deletion of *Plxnd1* during thymocyte development, expression of *Cre* recombinase from the *pKeratin14* promoter (27) in a second resulted in deletion of *Plxnd1* in thymic epithelial cells (TEC), and expression of *Cre* recombinase from the *pTek* promoter (28) in a third resulted in deletion of *Plxnd1* in endothelial cells. Using these mouse models, we have determined that loss of plexinD1 expression in thymocytes *per se* leads to the aberrant migration and cortical retention of CD69^+^ DP cells. On the other hand, ectopic subcapsular medullary formation results from loss of plexinD1 expression on the endothelial cells involved in vascular angiogenesis. Our results provide functional insight into the interplay of angiogenesis, thymocyte maturation, and thymic epithelial cell development in orchestrating T lineage differentiation.

## Materials and Methods

### Antibodies and reagents

AnnexinV-FITC, anti-FcγR (2.4G2), anti-CD69-FITC, anti-CD25-PE-Cy7, anti-CD44-APC-Cy7, anti-CD8α-FITC, and anti-CD4-APC were obtained from BD-Pharmingen (San Jose, CA, USA). Anti-ESAM-FITC and anti-MHCII (clone M5/114.15.2; anti-I-A/I-E) was obtained from eBioscience. ER-TR5 was provided by Dr. W. van Ewijk (Leiden University Medical Center, Netherlands), UEA1-FITC was obtained from Sigma-Aldrich. TROMA1 (anti-Keratin8 mAb) clone was obtained from Developmental Studies Hybridoma Bank (Iowa City, IA, USA). Sema3E-Fc was produced as described previously (15); note that the Fc is a mouse γ2c isotype. PE-conjugated F(ab′) anti-mouse γ2c heavy chain and IgG_2c_ control antibody was obtained from Jackson Immunoresearch (West Grove, PA, USA). 145.2C11 mAb was purified directly from hybridoma culture media using Gammabind Plus (GE HealthCare, NJ, USA) and dialyzed against PBS and adjusted to a final concentration of 2 mg/ml.

### Flow cytometry

Cell numbers were enumerated using a C-chip hemocytometer (NanoEntek, Korea). In general, single cell suspensions (2 × 10^6^ total) were blocked with 2.4G2 Ab and stained with anti-CD4-APC mAb, anti-CD8α-FITC, anti-CD25-PE-Cy7, anti-CD44-APC-Cy7 mAb, and purified sema3E-Fc (5 μg/ml) for 15 min. After washing with PBS, the cells were stained with anti-mouse IgG_2c_-PE to detect bound sema3E-Fc for an additional 15 min. After washing with PBS, the cells were resuspended in PBS and analyzed as described previously (15).

### Apoptosis analysis

One million total thymocytes were stimulated for up to 72 h on plates pre-coated with anti-CD3ε mAb (clone 145.2C11). After incubation, the cells were harvested and stained with anti-CD4-APC/anti-CD8α-PE/anti-TCRβ-FITC mAbs and analyzed by flow cytometry. The percentage viable DP thymocytes relative to input at *t* = 0 was then determined.

### Histology, immunohistology, and confocal microscopy

All the staining procedures were performed as described previously (15). Histological sections were stained using a standard hematoxylin and eosin procedure. In general, adjacent cryosections from thymi of 3- to 4-week-old WT, D1ThyCKO, D1EpCKO, and *Sema3e*^−/−^ mice were stained with the anti-CD4-APC/anti-CD8α-TRITC mAbs for delineation of cortex from medulla and with anti-CD69-FITC, and MHCII-FITC or UEA1-FITC as required. ER-TR5 (rat IgM) was visualized using anti-rat IgM-FITC. Images were obtained using a Zeiss LSM700 confocal laser-scanning microscope with 40× oil lens for objective lens (Carl Zeiss MicroImaging, NY, USA). H&E staining images were taken using a Nikon Eclipse Ti-S microscope with 4× dry lens for objective lens (Nikon Instruments, Inc., NY, USA). In every case, images were taken in monochrome to maximize resolution and then digitally recolored prior to merging of images. For identification of Pecam1 on developing endothelial structures, thymi from newborn WT and D1EnCKO mice were collected and fixed in 10% formalin overnight. Paraffin sections prepared from the fixed thymi were stained with anti-Pecam1 antibody and, after washing the sections were mounted with DAPI counterstaining the nuclei. In these images, high density nuclear DAPI staining corresponded to cortical regions and lower density staining corresponded with medullary regions.

### Mice and fetal thymi transplantation

*pLck-Cre, pKeratin14-Cre*, and *pTek-Cre* mice were purchased from Jackson Laboratory. The genotyping primers were synthesized by Eurofins. PCR reactions for detection of *Cre* gene detection were performed as recommended by the Jackson Laboratory. Each Cre strain was backcrossed onto *Plxnd1*^flox/flox^ background. Since the D1EnCKO mice were neonatal lethal, D1EnCKO or WT fetal thymi were prepared at embryonic day 16.5–17.5 and kept in sterile cold PBS. At this age, WT thymic lobes are fused to form one thymus with two lobes whereas D1EnCKO thymic lobes are not fused. After anesthetizing recipient mice using ketamine and xylazine, fetal thymi were transplanted under the renal capsule through a 0.5 cm cut in the skin. For WT transplants serving as controls, the complete thymus with two lobes was transplanted whereas for the D1EnCKO transplants, a single lobe was transplanted. After transplantation, the skin portal was closed using sutures. Following surgery, the cages with mice were warmed using hot packs until the mice recovered consciousness. Four weeks later, the thymi developing under the kidney capsule kidney were collected and immersed in OTC for cryosectioning. All the animal experiments were performed under animal protocols approved by Dana–Farber Cancer Institute and Harvard Medical School Animal Care and Use Committee Institutional Review Boards.

### Western blotting

Protein extract from total thymocytes was prepared with IP buffer [1% NP40, 50 mM Tris-HCl, pH 7.4, 150 mM NaCl, plus protease inhibitor cocktail (Roche, Germany)]. Lysate protein concentrations were determined using the BCA Protein Assay Kit (Pierce, IL, USA). Total lysate proteins were separated by SDS-PAGE (10%), transferred to PVDF, blocked overnight using TBS-T containing BSA (1%), blotted with anti-plexinD1 antisera (Sigma–Aldrich, MO, USA) followed by anti-rabbit-HRP secondary antibody (Invitrogen, CA, USA), and detection by chemiluminescence (ECL system; Amersham, VA, USA).

### Statistical analysis

Data are presented as mean values ± one standard error (SE). P values were calculated using Student’s *t*-test.

## Results

### Generation of mice with a thymocyte-specific *PlxnD1* CKO (D1ThyCKO)

Given that plexinD1 is operative in multiple developmental processes as described above, the T lineage autonomous effects of the germline *Plxnd1* KO contributing to the previously observed thymic phenotype versus non-T cell lineage cell expression of *Plxnd1* remained to be determined (15). Accordingly, a thymocyte-specific *Plxnd1* CKO mouse strain, termed D1ThyCKO, was created. To this end, *Plxnd1*^flox/flox^ mice generated with *loxP* sequences flanking the first exon encoding the transcription initiation site and 5′ sequence encoding the sema domain of the *Plxnd1* allele (14) were backcrossed with B6 mice multiple times (*n* > 10) to establish a B6 background. Next, these B6 *Plxnd1*^flox/flox^ mice were crossed with *pLck*-Cre transgenic B6 mice expressing a Cre recombinase under the control of the T cell-specific *pLck* promoter. Finally, offspring of these mice were intercrossed to yield the D1ThyCKO animals.

**Figure 1:**
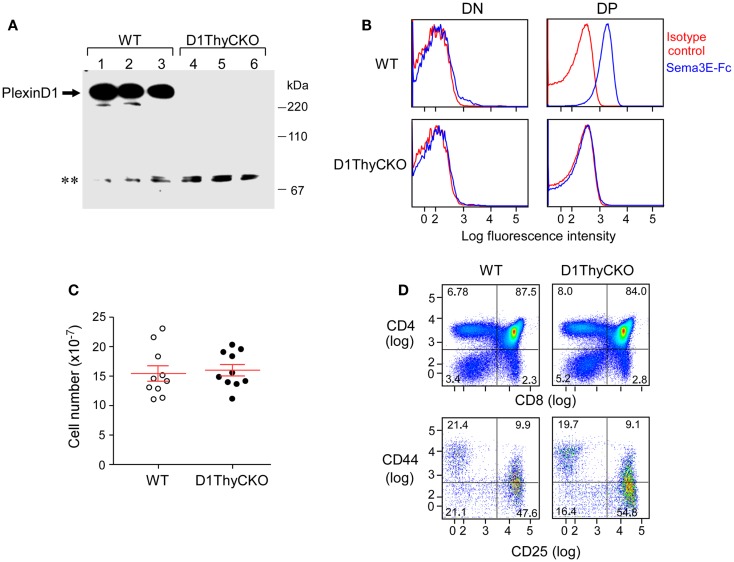
**PlexinD1 is not expressed in D1ThyCKO thymocytes but developmental progression is normal**. **(A)** PlexinD1 was detected in total thymocyte lysates from 3- to 4-week-old WT or D1ThyCKO mice (three mice/strain) by Western blotting. The lower bands (**) are non-specific bands common to both WT and D1ThyCKO thymocyte extracts and do not represent plexinD1 (representative of four independent experiments). **(B)** PlexinD1 detected by binding of sema3E-Fc (blue line) or control IgG2c (red line) after gating DP, CD4SP, CD8SP, and DN populations based on CD4 and CD8α expression (representative of six independent experiments). **(C)** Total thymocytes/thymus from WT or D1ThyCKO animals (*n* = 10 for each strain; *P* = 0.737, not significant). **(D)** Thymocyte differentiation in WT and D1ThyCKO mice. The percentage representations of DN (CD4^−^CD8^−^), DP (CD4^+^CD8^+^), CD4SP (CD4^+^CD8^−^), and CD8SP (CD4^−^CD8^+^) are depicted (upper panels). The percentages of DN1 (CD44^hi^CD25^lo^), DN2 (CD44^hi^CD25^hi^), DN3 (CD44^lo^CD25^hi^), and DN4 (CD44^lo^CD25^lo^) are shown (lower panels). Results represent six independent experiments.

In contrast to germline *Plxnd1*^−/−^ KO animals, the D1ThyCKO mice were viable and healthy, despite elimination of plexinD1 expression during T cell development. Absence of plexinD1 protein was evident both from Western blotting analysis of thymus lysates from these animals (Figure 1A) as well as by FACS analysis of their DP thymocyte surface plexinD1 expression detected by specific binding of sema3E-Fc (Figure 1B). On the other hand, plexinD1 was readily identified in WT samples. Despite the loss of plexinD1 protein, D1ThyCKO thymic cellularity was identical to that of the WT, indicating that loss of plexinD1 does not impact overall thymocyte expansion (Figure 1C). Further, the major CD4^−^CD8^−^ double negative (DN), CD4^+^CD8^+^ DP, and CD4^+^ and CD8^+^ single positive (SP) thymocyte subpopulations (defined by CD4 and CD8 expression) as well as progression through the DN1-4 stages (defined by CD44 and CD25 expression) as shown in Figure 1D were equivalent in D1ThyCKO and WT mice. Given that *Plxnd1* gene transcripts and plexinD1 protein expression appear at the DP thymocyte level, it is of particular note that the steady state expansions of DP and SP thymocytes in both mice are comparable.

As shown in Figure S1B in Supplementary Material, CD69^+^ expression was indistinguishable on WT and D1ThyCKO DP cells, suggesting that an equivalent number of thymocytes had undergone positive selection. In addition, the capacity of DP thymocytes to undergo apoptosis induced by anti-CD3ε mAb *in vitro* was identical (Figure S1A in Supplementary Material). Further, there were no differences between WT and D1ThyCKO in transwell migration assays responding to CXCL12 and CCL25 (Figure S2 in Supplementary Material). Thus, by analysis of comparative phenotype, signaling, and response to chemokines *in vitro*, WT and D1ThyCKO thymocytes appeared indistinguishable. Using global thymic transcriptome analysis, we observed that the only gene downregulated in D1ThyCKO thymocytes more than 10-fold is *Plxnd1* itself (data not shown). Accordingly, we attribute effects observed in the subsequent thymic translocation of D1ThyCKO cells *in vivo* during thymic organogenesis to loss of thymocyte plexinD1.

### DP thymocyte plexinD1 expression regulates cortical to medullary translocation after TCR engagement

**Figure 2:**
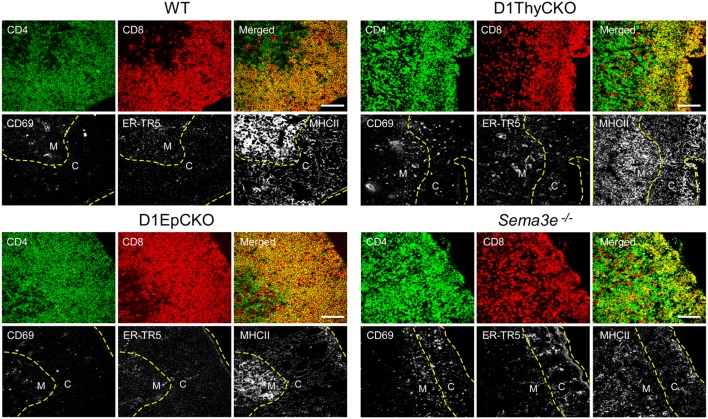
**CD69^+^ thymocytes are retained in the cortex in D1ThyCKO and *Sema3e*^−/−^ thymus but track to the medulla in WT and D1EpCKO mice**. For each strain, thymic frozen sections were stained for CD4 (green) and CD8 (red) expression where the DP cells in the cortical regions appear yellow in merged images due to the co-expression of CD4 and CD8. The merged yellow DP signal was used to delineate the cortex (C) from the individual SP red and green signals in the medulla (M) and a yellow dashed line is inserted to indicate the apparent corticomedullary junction. A second yellow dashed line indicates the capsule position. CD69 is an activation marker expressed by positively selected thymocytes following TCR stimulation. ER-TR5 is considered a medullary marker (29) while MHCII (I-A/I-E) is preferentially expressed in medulla (30). Each figure is representative of three independent experiments. White bar in the merged images is 100 μm.

**Figure 3:**
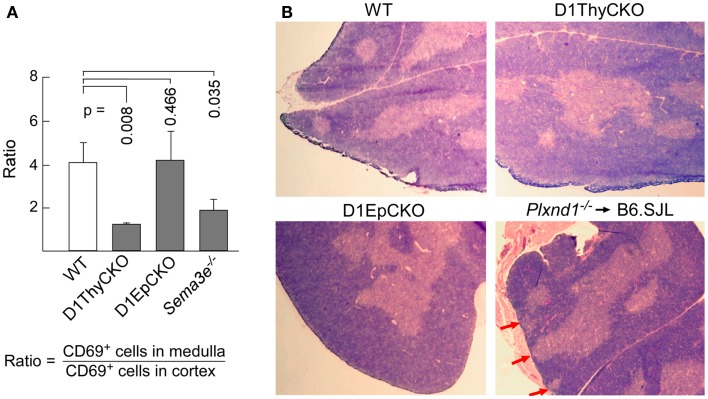
**Loss of plexinD1 disrupts cortical to medullary translocation of CD69^+^ DP thymocytes**. **(A)** The regional distribution of CD69^+^ cells in five separate frozen sections for each strain was determined based on FITC signal intensity within the regions defined by the apparent corticomedullary junction. After subtracting background signal, the ratio of fluorescence signal strength between the cortex and the medulla was calculated. A value >1 indicates stronger medullary presence on comparison with cortex. Error bars represent SEM. **(B)** Medullary structures were visualized by hematoxylin and eosin (H&E) staining of WT, D1ThyCKO, and D1EpCKO thymi as well as in thymus in irradiated animals repopulated with *Plxnd1*^−/−^ fetal liver. The red arrows in the *Plxnd1*^−/−^ section indicate regions of fused thymic medulla and capsule. Each figure is representative of three independent experiments.

We then ascertained whether the CD69^+^ post-selection D1ThyCKO thymocytes exhibited the same cortical retention pattern as we had observed previously using germline *Plxnd1*^−/−^ progenitors to repopulate Ly5 congenic animals post-transplant (15). Cortex and medulla were identified using anti-CD4 and anti-CD8 mAbs to define the medullary CD4 SP and CD8 SP thymocytes as well as merged CD4/CD8 signals to define cortical DP thymocytes (Figure 2). This delineation in the WT thymus was confirmed by the strong medullary expression of MHC class II and further confirmed by the medullary location for the ER-TR5 marker (29, 30). Of note, in the WT thymus the CD69^+^ post-selection thymocytes exhibit a medullary location while in the D1ThyCKO thymus, the CD69^+^ thymocytes are distributed not only in the medulla but also throughout the cortex. This phenotype is distinct from other lineage *Plxnd1* CKO mice to be described subsequently. Moreover, the ratio of medullary-localized to cortical-localized CD69^+^ post-selection thymocytes was enumerated (Figure 3A), confirming the cortical retention D1ThyCKO thymus.

These results independently confirmed our original observation that plexinD1 expression in developing thymocytes plays an important regulatory role in the cortical to medullary translocation of differentiating thymocytes following engagement of their TCRs by self-pMHC on TEC. However, in contrast to the results obtained with germline *Plxnd1* KO progenitor transplant reconstitution of the thymus, in the D1ThyCKO mice there is a completely normal thymic cortex and medullary demarcation on histological analysis (Figure 3B, upper panels) although at the level of CD4 and CD8 display, the separation between cortical DP and medullary SP thymocytes was less-well delineated, perhaps consequent to the differentiation within the cortex of mislocalized CD69^+^ cells (Figure 2), as previously observed with the germline *Plxnd1* KO mice (15). Furthermore, of note, the ER-TR5 and MHCII markers also disengage from the dominantly medullary location in WT thymus and now track with the cortically localized CD69^+^ thymocytes in D1ThyCKO thymus (Figure 2). Quantitation of the medullary to cortical ratio for ER-TR5 in wt thymus (2.97 ± 0.35; Figure S4 in Supplementary Material) supports its utilization as a relative medullary marker; likewise, the medullary to cortical ratio for MHC class II (8.37 ± 0.82) also supports its utilization as a medullary marker. In the D1ThyCKO thymus, however, the medullary to cortical ratio for both ER-TR5 and MHC class II was significantly reduced on comparison with wt thymus (1.39 ± 0.14 and 2.58 ± 0.54, respectively; Figure S4 in Supplementary Material). UEA1, also considered a medullary marker (31), does not segregate with ER-TR5 and MHCII in the D1ThyCKO mice (Figure S3 in Supplementary Material), instead remaining medullary in location. The alteration in ER-TR5 and MHCII distribution in association with CD69^+^ thymocytes suggests possible crosstalk between developing thymocytes and TEC. Interestingly, this disengagement of ER-TR5 from medullary structures and association with CD69^+^ cortically located DP thymocytes is recapitulated in the developing thymus of *Sema3e*^−/−^ mice (Figure 2). Hence, this phenotype is a result of the direct disruption of sema3E-plexinD1 signaling in thymocytes, since plexinD1 is normally expressed in the *Sema3e*-null tissues (32).

### While epithelial specific Plxnd1 CKO (D1EpCKO) is without thymic phenotype, transient stimulation of immature (pre-SP) thymocytes alters TEC developmental programing and initiates medullary formations

Since D1ThyCKO lacked any medullary abnormality detected by histology, we next examined whether loss of plexinD1 in the epithelial cell population may account for the aberrant medullary phenotype observed by germline *Plxnd1*^−/−^ progenitor repopulation of B6.SJL thymi. Here we used *pKer14-Cre*^+^ *Plxnd1*^flox/flox^ (D1EpCKO) mice where activation of *pKer14* during the bipotential TEC progenitor stage (33) will lead to deletion of *Plxnd1* in descendant cTEC and mTEC. We found that the D1EpCKO mice were viable and exhibited normal thymic development and organization (Figure 2), histology (Figure 3B), and thymocyte development (data not shown). The ratio of medullary to cortical CD69^+^ thymocytes was ∼4, directly comparable to that for WT CD69^+^ thymocytes migrating to the medulla (Figure 3A). Reinforcing the observed normal medullary development, in contrast to what was observed in D1ThyCKO mice, the ER-TR5 signal in D1EpCKO thymus localized with UEA1 in medullary regions comparable to that observed in WT thymus (Figure S3 in Supplementary Material). Thus epithelial loss of plexinD1 neither affected cortical to medullary migration of CD69^+^ thymocytes nor medullary development.

Our results imply that CD69^+^ thymocytes are responsible for the development of ER-TR5^+^ thymic signal and that ER-TR5 is a marker linked to CD69^+^ thymocyte position as opposed to medullary locale *per se*. This further implies that maturing DP thymocytes following TCR stimulation may play a role in initiating medullary development, a function previously ascribed exclusively to mature SP T cells (34). It has been reported previously that expression of αβ TCR transgenes, even on a non-selecting background, may induce ER-TR5^+^ medulla development suggesting a role for αβ TCR induction of thymic medullary structures in the absence of TCR activation (35). Since *Tcrb*^+/+^*Rag2*^−/−^ mice do not develop SP thymocytes or thymic medullary structures (36, 37), constitutive pre-TCR expression is insufficient for induction of medulla. In such mice, T cell development is arrested at the pre-TCR DP stage, but we reasoned that the pre-TCR and/or incomplete surface CD3 heterodimers (38) on these thymocytes might be stimulated via intravenous administration of anti-CD3ε mAb (clone 145.2C11) that rapidly enters the thymus (39).

**Figure 4:**
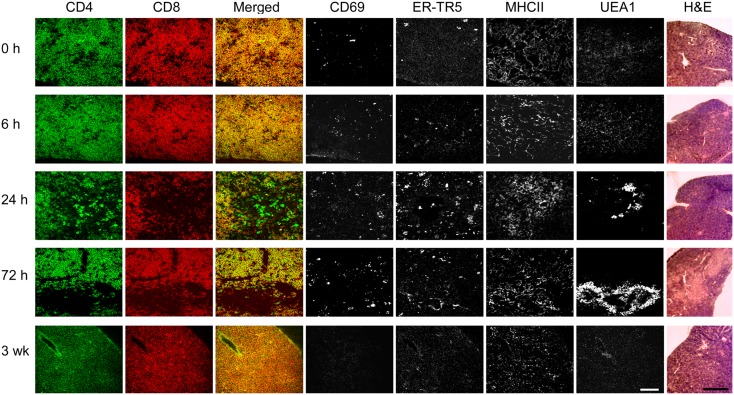
**pre-TCR DP thymocytes can induce medullary formation on activation**. At time points from 0 h to 3 weeks, cryosections from thymi of *Tcrb*^+/+^*Rag2*^−/−^ mice injected at 0 h with anti-CD3ε mAb were prepared. Adjacent cryosections were stained with anti-CD69 to identify activated DP thymocytes, anti-CD4, and anti-CD8 to detect DP and SP thymocytes and differentiate cortical from developing medullary structures, and with ER-TR5, UEA1, and anti-MHCII (I-A/I-E) to identify putative medullary elements. Five slides of each thymus were H&E stained. This experiment was repeated with identical results. White bar in the merged images represents 100 μm and the black bar in the H&E panels represent 5 mm.

To determine whether the DP thymocytes could be activated and induce medullary development, anti-CD3ε antibody was injected into *Tcrb*^+/+^*Rag2*^−/−^ mice and thymi were collected at 0, 6, 24, and 72 h to assess early activation and medulla development. A final analysis was done at 3 weeks post-injection to determine persistence and/or evolution of phenotypic changes. As shown in Figure 4, by 24 h post-2C11 injection, distinct regions containing CD4 and CD8 SP thymocytes were observed with extensive representation of the ER-TR5 medullary marker, MHC class II expression and UEA1 expression together with increased representation of CD69^+^ thymocytes. By 72 h, these developmental changes had led to clear histological delineation of cortical and medullary regions but the representation of SP cells was diminished. The majority of cells were DP thymocytes and the ER-TR5 signal was considerably weaker while the UEA1^+^ signal, correlating well with areas of poor DP thymocyte representation, became even stronger. This latter result suggests that ER-TR5 discriminates early signals delivered by CD69^+^ DP thymocytes from those inducing development of UEA1^+^ mTEC, as observed for the disengagement of ER-TR5 signal from that of UEA1 in the D1ThyCKO and *Sema3e*^−/−^ thymi where CD69^+^ thymocytes were retained in the cortex (Figure 2 and Figure S3 in Supplementary Material). In contrast, by 3 weeks after the single anti-CD3ε mAb injection, both ER-TR5 and UEA1 signals had diminished to almost undetectable levels. All indications of medullary formation had disappeared including histological demarcation of cortex and medulla, leading to a set of images indistinguishable from those at time 0 h. Of note, in every instance, the developing corticomedullary junction was proximal to pre-existing larger vasculature defined by Endothelial cell Selective Adhesion Molecule (ESAM) as shown in Figure S5 in Supplementary Material (40). One possibility is that 2C11 distribution from the vasculature is greatest at these sites to most optimally trigger CD3 crosslinking on developing thymocytes.

### Endothelial cell plexinD1 expression impacts thymic medulla location through regulation of angiogenesis: Analysis of D1EnCKO mice

Although we had observed ectopic medullary formation in thymi of B6.SJL mice repopulated *in vivo* by *Plxnd1*^−/−^ fetal liver cells, no such abnormality was observed in D1ThyCKO or D1EpCKO mice where plexinD1 expression was specifically eliminated in thymocytes or epithelial cells, respectively. We concluded that plexinD1 must be operating in a distinct non-hematopoietic, non-epithelial lineage to impact thymic medullary developmental formation. In this regard, it is well established that within the fetal liver there are stem cells not only with lymphoid and myeloid potential but also capable of differentiating into hepatocytes and biliary epithelial cells (25, 41, 42). Further, the fetal liver tissue expresses endothelial progenitor cells with angiogenic potential that may contribute to thymic vascular development (23, 24). Since plexinD1 and sema3E play a seminal role in controlling vascular endothelial branching during development, the possibility that *Plxnd1*^−/−^ endothelial stem cells delivered in the fetal liver inoculate may have influenced ectopic subcapsular medullary formation in our earlier report required further elucidation (15). To address whether plexinD1 expression on endothelial cells contributes to vasculature pattern formation, thereby supporting proper medullary formation, we crossed *Plxnd1*^flox/flox^ mice and *pTek*(*Tie2*)-*Cre* transgenic mice expressing the *Cre* transgene in an endothelial-specific manner. The resulting *pTek*-Cre^+^*Plxnd1*^flox/flox^ (D1EnCKO) conditional mice manifest a defect in separation of the aorta and the pulmonary artery as previously reported and did not survive the neonatal period (11, 14). Furthermore, the thymic lobes failed to fuse, instead forming two separate thymic lobes on both sides of the truncus artery as we observed for germline *Plxnd1*^−/−^ mice (15).

**Figure 5:**
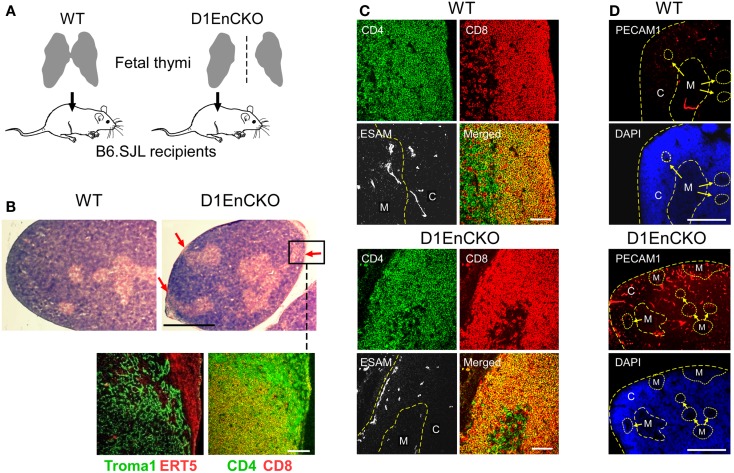
**PlexinD1 on thymic endothelial cells regulates the distribution of thymic medullary formations**. **(A)** Schematic for analysis of D1EnCKO cells on thymus development. Fetal thymi harboring WT (both fused lobes transplanted) or D1EnCKO (single lobe transplanted) endothelial cells were allowed to develop under the recipient’s renal capsule. All cell populations in the transplanted D1EnCKO thymus other than endothelial cells expressed plexinD1 appropriately as did all cells in the normal recipients. **(B)** Red arrows in H&E stained panels indicate regions of medulla fused with the thymic capsule. A frozen section corresponding to the inset region in the D1EnCKO panel was stained for keratin 8 highly represented in cTEC using the Troma1 antibody (56, 57) and for ER-TR5 representing medullary development. Cortical-medullary demarcation was confirmed by the relative representation of merged CD4^+^CD8^+^ signal for DP cells and SP CD4 and CD8 cell development in the subcapsular region. Result is representative of three independent transplantation experiments. White bar in the confocal image represents 100 μm and in the H&E stained image represents 5 mm. **(C)** Cryosections of WT and D1EnCKO thymus were stained with anti-CD4-APC, anti-CD8α-TRITC, and anti-ESAM mAb-FITC. Dashed yellow line follows the corticomedullary junction; in the D1EnCKO panel, a second dashed yellow line to the left indicates the ESAM^+^ cells in the subcapsullar zone. C, cortex; M, medulla. Result is representative of three independent transplantation experiments. White bar in the merged images are 100 μm. **(D)** Pecam1 staining to identify developing vascular endothelial structures. High density nuclear staining by DAPI delineates cortical regions and lower density nuclear staining correlates with medullary structures. C, cortex; M, medulla. The corticomedullary junctions and capsules are identified by dashed yellow lines. White bar in the images is 5 mm.

To overcome the neonatal lethality of D1EnCKO mice, we transplanted excised fetal thymi under the renal capsule of WT B6.SJL recipient mice. This procedure allows the thymus carrying *Plxnd1*^−/−^ endothelial cells to complete organogenesis (Figure 5A). After 3 weeks, the thymi with attached kidney were excised and cryosections prepared for histological analysis. The apposition of medulla to the thymic capsule was clearly observed histologically in the D1EnCKO thymus explant (Figure 5B). Analysis of sequential cryosections demonstrated that these medullary structures contained predominantly CD4 SP thymocytes and stained with the ER-TR5 medullary marker (Figure 5B inset).This medullary structure is distinct from the cortical region otherwise marked by Troma1 (anti-cytokeratin 8), a cTEC-expressed protein. In contrast, CD4 SP thymocytes are not present in the cortical subcapsular region of WT thymus where the merged signal of CD4 and CD8 on DP thymocytes dominated (Figures 2 and 5C).

**Figure 6:**
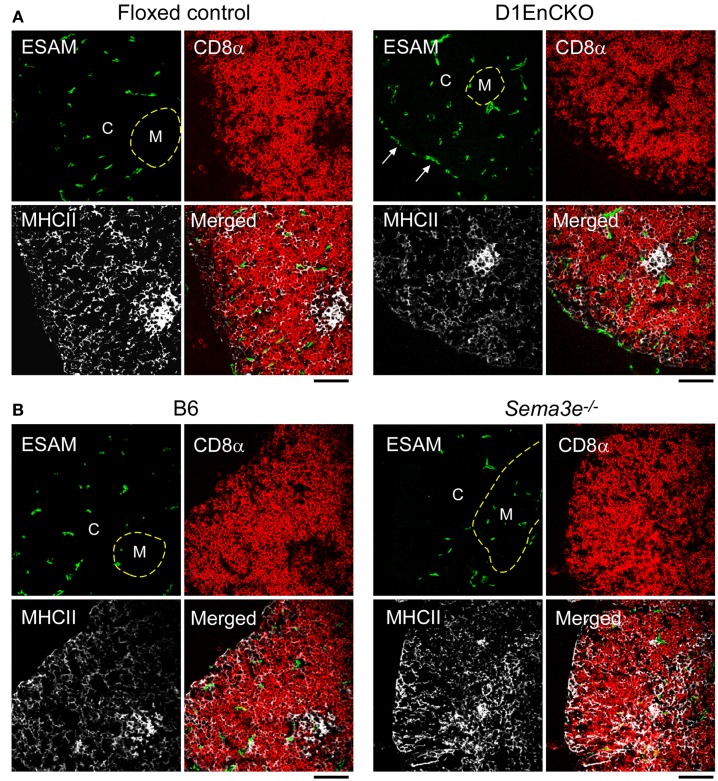
**Differential development of ESAM^+^ blood vessels in WT, D1EnCKO and *Sema3e*^−/−^ thymi**. Thymic cryosections of the indicated control mice and D1EnCKO **(A)** and *Sema3e^−/−^* **(B)** mice were stained with anti-MHCII-APC, anti-CD8α-TRITC, and anti-ESAM mAb-FITC. Dashed line follows the corticomedullary junction. The result is representative of one of three independent experiments. The black bar below each merged image represents 100 μm and is applicable to every panel in each block. C, cortex; M, medulla.

To next determine whether these abnormal subcapsullary medullary formations were related to vascular development, we examined the expression of the vascular ESAM marker for significant differences between the WT and D1EnCKO thymi. In WT thymus, the ESAM signal was detected at highest levels on both sides of the corticomedullary junction with little representation in the outermost cortex (Figure 5C and Figure S6 in Supplementary Material). In contrast, in the D1EnCKO thymus the ESAM signal was broadly spread throughout the cortical region with strongest representation in the subcapsular area and less of a medullary presence (Figure 5C). The regulatory functions of plexinD1 for vascular endothelial branching are initiated by binding its ligand sema3E, leading us to examine whether a similar pattern of ESAM^+^ vascular structures was present in the *Sema3e*^−/−^ thymus. As shown in Figure 6 and Figure S6 in Supplementary Material, unlike the pattern in the D1EnCKO thymus, the ESAM signal was broadly distributed throughout the *Sema3e*^−/−^ thymus without subcapsular representation implying that some functionality of plexinD1 in thymic vascular pattern formation is apparently sema3E-independent. Certainly, no difference was observed in the distribution of vascular ESAM^+^ signal around the corticomedullary junction in WT and D1ThyCKO thymus (Figure S6 in Supplementary Material), implying the ectopic medullary formations may be related to the effects of plexinD1 and sema3E upon thymic vascular bed development. Conversely, the distribution of CD69^+^ thymocytes in the D1EnCKO thymus clearly shows a strong medullary presence of CD69^+^ cells. Although there is collection of CD69^+^ cells around the corticomedullary junction, this did not translate into an overt retention of CD69^+^ cells in the cortex (Figure S7 in Supplementary Material) and a normal medullary structure with appropriate localization of MHC class II, ER-TR5, and mTEC-expressed UEA1 and a clearly demarcated corticomedullary junction (Figure S7 in Supplementary Material). Consequently, loss of plexinD1 on the thymic endothelial cell population does not affect CD69^+^ thymocyte translocation from the cortex to the medulla but it does seem to alter cortical and medullary vascular development in a sema3E-independent manner.

To demonstrate that the development of D1EnCKO fetal thymus under recipient renal capsules properly represented the development ongoing in D1EnCKO, we analyzed thymi from newborn D1EnCKO mice using anti-Pecam1 (Platelet Endothelial Cell Adhesion Molecule-1, CD31) mAb that identifies vascular endothelial cells including those in newly formed vasculature (43) (Figure 5D and Figure S8 in Supplementary Material). In WT thymi the Pecam1 signal was detected in a punctuate distribution throughout the thymus while in D1EnCKO thymi the Pecam1 distribution was much more dense, with a 5.03 ± 0.78-fold increase in endothelial cell density over WT. Pecam1 expression was particularly strong in cortical areas and the sections contained many ectopic medullary structures, several of which appeared to be fused with the capsule. Thus the histological evidence for ectopic medullary formation in the *Plxnd1*^−/−^ thymi can be definitively attributed to the expression of plexinD1 in thymic endothelial cells and their essential function in thymic vascular development.

## Discussion

Through cell-type specific deletion of plexinD1 during thymic organogenesis, we have dissected the involvement of plexinD1 and its ligand, sema3E, in thymic development dependent upon thymocyte, epithelial, and vascular endothelial cell crosstalk for proper organ pattern formation. We demonstrate that expression of plexinD1 during the DP stage of thymocyte differentiation is a necessary prerequisite for the orderly cortical to medullary translocation that follows positive selection. In the absence of thymocyte plexinD1, despite appropriate expression on all other cells in the developing thymus, the activated CD69^+^ cells do not translocate toward the medulla, instead largely remaining within the cortex. Further, those mislocalized CD69^+^ cells initiate developmental programing in local TEC cells in the cortex that induces the ER-TR5 signal, a well characterized though molecularly undefined marker of the mTEC environment and used to define medullary locales. On the other hand, loss of plexinD1 in epithelial cells, either cTEC or mTEC, was unremarkable and hence of no consequence for any of the parameters monitored in the current study.

In contrast, loss of plexinD1 in the thymic endothelial progenitor population led to distinct reorganization of thymic vasculature that was associated with ectopic medullary formation in the cortex and a medulla topology characterized by fusion with the capsule. We observed this phenotype previously in thymi repopulated by *Plxnd1* null cells (15). Thus thymic angiogenesis attributable to endothelial progenitors in these *Plxnd1*^−/−^ CKO mice and earlier germline *Plxnd1*^−/−^ KO progenitors determines the locale of medulla formation. We further show that currently accepted markers of mTEC are dynamically regulated since the cortically localized CD69^+^ DP thymocytes in D1ThyCKO were associated with increased cortical representation of ER-TR5 and MHCII markers, but not UEA1. The latter remained associated with the pre-formed, i.e., established, medullary structures. Finally, reinforcing the importance of the DP thymocytes in contributing to the initial although transient establishment of medullary formation, we demonstrate that DP activation in a *Tcrb*^+/+^*Rag2*^−/−^ thymus results in development of medullary regions populated with SP thymocytes despite a genetic defect that precludes TCR expression and allows only pre-TCR to be displayed on the surface of DP thymocytes.

Our results clearly point to a dynamic interplay of thymocyte differentiation and vascular localization. Both processes are impacted by plexinD1 expression in independent cellular compartments, with consequent effects upon cTEC and mTEC function and medullary formation in turn effecting thymocyte differentiation. Since the thymocytes migrate along a path governed by differentiation-determined expression and function of chemokine receptors (44, 45), it is reasonable to conclude that blocks on maturation of thymocytes, exemplified here by loss of plexinD1, will lead to significant aberrations in thymus morphology. This notion is supported by results indicating that cTEC do not develop in mice where the DN1 → DN3 transition is defective (46) while a block on SP thymocyte development leads to absence of medullary formation, a state that can be altered by thymic influx of passively transferred SP T cells in SCID mice (34, 35). Collectively, such observations argue that mature SP cells are required for sustained medullary formation. In addition, we show here that anti-CD3ε mAb triggered DP thymocytes can initiate differentiation of medullary structures, even if located in ostensibly “cortical” areas. Likewise, blocks in the orchestrated trafficking of differentiating thymocytes also may lead to aberrations in thymic morphology. For example, deficiency of CCR7 or of its ligands CCL19/CCL21 results in thymocytes maintaining a cortical position associated with ectopic medullary structure formation in the subcapsular zone and spontaneous autoimmunity with increased titers of circulating antibodies that lead to IgG deposition in renal glomeruli (47, 48).

The DP thymocytes constitute the major population in the thymic cortex and we previously identified plexinD1 as a key regulator of DP thymocyte chemokine responses (15). In WT thymus repopulated by *Plxnd1*^−/−^ thymic precursors, we noted not only the cortical retention of CD69^+^ (activated) DP cells confirmed in this report in the D1ThyCKO mice, but also ectopic medullary formations lying under the capsule. In the context of plexinD1’s key role in controlling vascular development and branching (11, 20), we investigated the possibility that the fetal liver containing early thymocyte progenitor cells used to repopulate the irradiated thymi contained *Plxnd1*^−/−^ endothelial precursors resulting in aberrant thymic vasculature formation. As noted previously, the development of the vascular bed in the thymus plays a key role in organizing the medullary epithelial cell compartment (49). Since D1EnCKO was neonatal lethal, we transplanted the D1EnCKO thymi under the recipient renal capsule to follow development. Under this circumstance, the abnormal fusion between thymic capsule and medulla was observed, demonstrating that blood vessel patterning directly affects the medulla location in thymus. Unlike the vasculature of the WT thymus, ESAM^+^ blood vessels exist under the thymic capsule of D1EnCKO mice. In thymi lacking sema3E, however, a completely different phenotype was observed; ESAM^+^ vessels were not observed under the thymic capsule but instead found broadly distributed throughout the thymic cortex.

This discordance suggests that either plexinD1 may respond to other thymic ligands distinct from sema3E, or that plexinD1 may contribute to intracellular regulatory pathways even in the absence of extracellular sema3E-mediated stimulation. In addition to the regulation of plexinD1 expression by VEGF mentioned above, the signaling for repulsion of developing neurites mediated by sema3E signaling through plexinD1 is mitigated or reversed by the ectodomain of neuropilin-1 (32). Of note, we observe no evidence for any direct interaction of neuropilin-1 ectodomain with either sema3E, plexinD1, or the sema3E-plexinD1 complex (data not presented), suggesting regulation of plexinD1 function by alternative signaling cascades mediated by Nrp1.

The apparently normal medullary structure and cortical-medullary demarcation in D1ThyCKO thymus by histological analysis in the context of a disorderly corticomedullary junction by confocal microscopy analysis point to a disengagement of medullary processes required for normal thymic function. In this report, we used ER-TR5 with or without UEA1 as another medullary marker in addition to MHC class II expression (29–31, 50). In general, the MHC class II expression correlated well with that of ER-TR5. In the D1ThyCKO thymus, however, UEA1 remained associated with the histologically defined medulla while ER-TR5 uncoupled from this signal and localized with the cortically retained CD69^+^ DP thymocytes. Consequently, we propose that ER-TR5 is not a medullary marker but should be considered an epithelial cell marker induced by juxtaposition to developing activated T lymphoid cells. This scenario seems more probable than the suggestion that mTEC are rapidly migrating from the established medulla into the cortex, although such a possibility must be formally excluded in future studies.

An interesting corollary is that lack of plexinD1 on the DP thymocytes does not inhibit the differentiation of the thymocytes but only inhibits their migration. The involvement of plexinD1 as a regulator of migration rather than differentiation is supported strongly by similar functionality controlling migration not only of thymocytes but also B cells, macrophages, osteoblasts, prostate cancer cells, as well as colorectal tumor cell and melanoma cell line metastasis in models *in vivo* (15, 16, 51–55). Control of migration has many facets, however, and may represent a regulation of adhesion through integrins rather than migration itself. Certainly, in transwell migration assays in the absence of potential interacting extracellular matrix components, we observe no effect of plexinD1 loss upon thymocyte responses to either CXCL12 or CCL25 (Figure S2 in Supplementary Material) and we have recently identified a direct role for plexinD1 independently as well as in a sema3E-dependent manner for regulating integrin adhesion (manuscript submitted). The additional role of plexinD1 in regulating thymic endothelial cell angiogenesis clearly has a direct impact upon the location of medullary formations. In this respect, it is of note that regional variations in thymic oxygenation are of critical importance for thymic structure as well as possible influence upon selection processes during thymocyte development (17–19).

## Conclusion

The results presented herein represent a dissection of the simultaneous activities of the transmembrane receptor, plexinD1, functioning in disparate developmental processes mediated by different cellular compartments to regulate thymic organogenesis. PlexinD1 loss has a profound effect upon CD69^+^ positively selected DP thymocyte trafficking. The mislocalization of this thymocyte subset in the absence of gross alterations in thymic morphology impacts markers previously believed to delineate the medullary compartment. Meanwhile, loss of plexinD1 in vascular endothelial cells during thymic development leads to a distinct rearrangement of the thymic vascular structure that is clearly related to gross histological abnormalities in thymic morphology. In general, plexin signaling activity has been viewed as relatively passive in the absence of ligand. Comparison, however, of the thymus phenotypes of the WT, D1EpCKO, D1ThyCKO, and *Sema3e*^−/−^ null mice lays the groundwork for examination of plexinD1 signaling and functionality at the molecular level in both a sema3E ligand-independent and sema3E-dependent fashion.

## Author Contributions

Young I. Choi, Jing Tan, Jingang Gui, and Manvendra K. Singh performed all of the experiments. Young I. Choi, Jonathan S. Duke-Cohan, Jonathan A. Epstein, and Ellis L. Reinherz designed the experiments and analyzed the data. Jonathan S. Duke-Cohan, Young I. Choi, and Ellis L. Reinherz wrote the paper.

## Conflict of Interest Statement

The authors declare that the research was conducted in the absence of any commercial or financial relationships that could be construed as a potential conflict of interest.

## Supplementary Material

The Supplementary Material for this article can be found online at http://www.frontiersin.org/Journal/10.3389/fimmu.2013.00392/abstract

Figure S1 shows that anti-CD3ε-induced apoptosis is identical for WT and D1ThyCKO thymocytes, and that within the D1ThyCKO thymus, the number of developing CD69^+^ DP thymocytes is not significantly different to that in WT thymus thus any alteration in thymic localization represents a true positional shift in the DP population. Figure S2 depicts the identical responses of WT and D1ThyCKO cells to CXCL12 and CCL25 chemokines, supporting the proposal that mislocalization of the latter population is due to alteration in guidance cue signaling rather than an intrinsic defect in migratory capability. Figure S3 is a direct supplement to Figure 2, showing the distribution of the medullary UEA1 markers in the same thymic sections described in Figure 2. Figure S4 quantifies the disengagement of cortical ER-TR5 and MHC class II signal from canonical medullary structures observed in Figure 2. Figure S5 shows the development of transient medullary structures next to ESAM+ blood vessels in *Tcrb*^+/+^*Rag2*^−/−^ mice injected with anti-CD3ε mAb. Figure S6 reveals the disruption of association of ESAM^+^ vessels with the corticomedullary junction in *Sema3e*^−/−^ thymus. Figure S7 shows normal cortical and medullary thymic marker localization in D1EnCKO thymus, even though some of the medullary structures may be embedded in the cortex and close to the capsule. Figure S8 demonstrates the dysregulation of vasculogenesis in D1EnCKO newborn thymus revealed by staining for endothelial cell Pecam1.

Figure S1 ***Plxnd1* deletion does not affect apoptosis of, or CD69 expression on, thymocytes following TCR stimulation**. **(A)** One million total thymocytes were stimulated with plate-coated anti-CD3ε mAb for the indicated time period. After incubation, the cells were harvested and stained with anti-CD4-APC/anti-CD8α-PE/anti-TCRβ-FITC mAbs and analyzed by flow cytometry. The %live DP population was normalized against %live DP population at 0 h. Error bars represent SEM. The result is representative of two independent experiments. **(B)** After stimulation of total thymocytes for the indicated time periods as in **(A)**, the cells were harvested and stained with anti-CD4-APC/anti-CD8α-PE/anti-CD69-FITC mAbs and analyzed by flow cytometry. %CD69^+^ DP thymocytes was calculated relative to total DP thymocytes. Error bars represent SEM. The result is representative of two independent experiments.

Figure S2 **CXCL12- and CCL25-mediated migration is normal in D1ThyCKO mice**. Migration assays were performed with CXCL12 and CCL25 as described previously using both chemokines at 1 μg/ml (15). After the incubation for 2 h, the cells migrating across the transwell membrane into the lower well were harvested and counted. White bars depict mock-treated (no chemokine) control experiments. Error bars represent SEM.

Figure S3 **UEA1 expression in plexinD1 conditional knockout and *Sema3e*^−/−^ thymus**. UEA1 expression in sections correlating to those depicted in Figure 2 in the main article. Note that the bulk of UEA1 signal is medullary in all cases. “M” defines medullary area, “C” defines cortical area; the dashed line represents the corticomedullary junction, and the solid line represents the thymic capsule.

Figure S4 **Quantitation of relative cortical and medullary ER-TR5 and MHC class II expression in wt and D1ThyCKO thymi**. The relative signals for cortical and medullary-localized fluorescence were determined by image analysis of six D1ThyCKO thymic sections and nine wt sections. For each marker, the color image was converted to a grayscale image, six to seven regions without apparent signal were selected to represent background and the relative intensity for identically sized regions within areas demarcated as cortex or medulla were determined using the ImageJ java suite. The D1ThyCKO medullary MHC class II representation was significantly reduced on comparison with wt (*P* < 0.005) as was the medullary ER-TR5 representation (*P* < 0.02). Mean ± SEM presented and compared using Student’s *t*-test.

Figure S5 **Co-localization of ESAM^+^ blood vessels and UEA1^+^ mTEC in thymi of *Tcrb*^+/+^*Rag2*^−/−^ mice injected with anti-CD3ε mAb**. Adjacent cryosections of each thymus were stained with the indicated Abs. anti-ESAM was used to visualize endothelial cells and UEA1 to visualize mTEC. The corticomedullary junction is indicated by the dashed line. The result represents two independent analyses. White bar in the ESAM panels is 100 μm and applies to all panels.

Figure S6 **ESAM^+^ blood vessel distribution is normal in D1ThyCKO mice**. The thymic cryosections of the indicated mice were stained as described in Figure 5C. The result is representative of three independent experiments. The dashed line follows the corticomedullary junction where the demarcation between cortex and medulla is based on the location of DP and SP thymocytes, respectively. ESAM is an endothelial cell-specific marker. White bar in merged image is 100 μm. C, cortex; M, medulla.

Figure S7 **Normal CD69^+^ thymocyte distribution in transplanted D1EnCKO mice**. Adjacent cryosections of D1EnCKO thymi transplanted under the B6.SJL renal kidney capsule were stained as described for Figure 2 and Figure S3. The cortex (C) and the medulla (M) were divided based on the location of DP and SP thymocytes. The left dashed line in each section follows the corticomedullary junction and the right dashed line depicts the thymic capsule. White bar in merged images is 100 μm. The figure is representative of two independent experiments. The graph depicts the enumeration of CD69^+^ cell density using the FITC signal intensity as described in Figure 3. Sections from three independent thymi were analyzed for each genotype (wt and D1EnCKO) and *P* values were calculated using Student’s *t*-test where the ordinate axis depicts the ratio of CD69^+^ cells in the medulla to CD69^+^ cells in the cortex. Error bars represent SEM. The *P* value of 0.34 is not statistically significant.

Figure S8 **Pecam1^+^ endothelial cells are increased in newborn D1EnCKO mice**. **(A)** were collected from WT (*n* = 2, “*Control*” in figure) and D1EnCKO (*n* = 3, “*Mutant*” in figure) newborn mice and fixed overnight in 10% formalin solution, then stained for Pecam1 (CD31). **(B)** The overall intensity/area was determined by image analysis for WT, and then both WT and D1EnCKO Pecam1 densities were expressed as a ratio of the WT values. Mean ± SEM is depicted. The white bar, applicable to all panels, represents 2 mm.

## References

[1] Kruger RP Aurandt J Guan KL Semaphorins command cells to move. Nat Rev Mol Cell Biol (2005) 6:789–800 10.1038/nrm1740 16314868

[2] Rohm B Rahim B Kleiber B Hovatta I Puschel AW The semaphorin 3A receptor may directly regulate the activity of small GTPases. FEBS Lett (2000) 486:68–72 10.1016/S0014-5793(00)02240-7 11108845

[3] Oinuma I Katoh H Negishi M Molecular dissection of the semaphorin 4D receptor plexin-B1-stimulated R-Ras GTPase-activating protein activity and neurite remodeling in hippocampal neurons. J Neurosci (2004) 24:11473–80 10.1523/JNEUROSCI.3257-04.2004 15601954 PMC6730355

[4] Oinuma I Ishikawa Y Katoh H Negishi M The semaphorin 4D receptor Plexin-B1 is a GTPase activating protein for R-Ras. Science (2004) 305:862–5 10.1126/science.1097545 15297673

[5] Tong Y Chugha P Hota PK Alviani RS Li M Tempel W Binding of Rac1, Rnd1, and RhoD to a novel Rho GTPase interaction motif destabilizes dimerization of the plexin-B1 effector domain. J Biol Chem (2007) 282:37215–24 10.1074/jbc.M703800200 17916560 PMC2655321

[6] Wang Y He H Srivastava N Vikarunnessa S Chen YB Jiang J Plexins are GTPase-activating proteins for Rap and are activated by induced dimerization. Sci Signal (2012) 5:ra6 10.1126/scisignal.2002636 22253263 PMC3413289

[7] Halloran MC Wolman MA Repulsion or adhesion: receptors make the call. Curr Opin Cell Biol (2006) 18:533–40 10.1016/j.ceb.2006.08.010 16930978

[8] Oinuma I Katoh H Negishi M Semaphorin 4D/Plexin-B1-mediated R-Ras GAP activity inhibits cell migration by regulating beta(1) integrin activity. J Cell Biol (2006) 173:601–13 10.1083/jcb.200508204 16702230 PMC2063868

[9] Sakurai A Jian X Lee CJ Manavski Y Chavakis E Donaldson J Phosphatidylinositol-4-phosphate 5-kinase and GEP100/Brag2 protein mediate antiangiogenic signaling by semaphorin 3E-plexin-D1 through Arf6 protein. J Biol Chem (2011) 286:34335–45 10.1074/jbc.M111.259499 21795701 PMC3190799

[10] Watakabe A Ohsawa S Hashikawa T Yamamori T Binding and complementary expression patterns of semaphorin 3E and plexin D1 in the mature neocortices of mice and monkeys. J Comp Neurol (2006) 499:258–73 10.1002/cne.21106 16977617

[11] Gitler AD Lu MM Epstein JA PlexinD1 and semaphorin signaling are required in endothelial cells for cardiovascular development. Dev Cell (2004) 7:107–16 10.1016/j.devcel.2004.06.002 15239958

[12] Torres-Vazquez J Gitler AD Fraser SD Berk JD Van NP Fishman MC Semaphorin-plexin signaling guides patterning of the developing vasculature. Dev Cell (2004) 7:117–23 10.1016/j.devcel.2004.06.008 15239959

[13] Kanda T Yoshida Y Izu Y Nifuji A Ezura Y Nakashima K PlexinD1 deficiency induces defects in axial skeletal morphogenesis. J Cell Biochem (2007) 101:1329–37 10.1002/jcb.21306 17477353

[14] Zhang Y Singh MK Degenhardt KR Lu MM Bennett J Yoshida Y Tie2Cre-mediated inactivation of plexinD1 results in congenital heart, vascular and skeletal defects. Dev Biol (2009) 325:82–93 10.1016/j.ydbio.2008.09.031 18992737 PMC2650856

[15] Choi YI Duke-Cohan JS Ahmed WB Handley MA Mann F Epstein JA PlexinD1 glycoprotein controls migration of positively selected thymocytes into the medulla. Immunity (2008) 29:888–98 10.1016/j.immuni.2008.10.008 19027330 PMC2615553

[16] Holl EK O’Connor BP Holl TM Roney KE Zimmermann AG Jha S Plexin-D1 is a novel regulator of germinal centers and humoral immune responses. J Immunol (2011) 186:5603–11 10.4049/jimmunol.1003464 21464091 PMC3771081

[17] Hale LP Braun RD Gwinn WM Greer PK Dewhirst MW Hypoxia in the thymus: role of oxygen tension in thymocyte survival. Am J Physiol Heart Circ Physiol (2002) 282:H1467–77 10.1152/ajpheart.00682.2001 11893584

[18] Biju MP Neumann AK Bensinger SJ Johnson RS Turka LA Haase VH Vhlh gene deletion induces Hif-1-mediated cell death in thymocytes. Mol Cell Biol (2004) 24:9038–47 10.1128/MCB.24.20.9038-9047.2004 15456877 PMC517905

[19] Bryson JL Griffith AV Hughes B III Saito F Takahama Y Richie ER Cell-autonomous defects in thymic epithelial cells disrupt endothelial-perivascular cell interactions in the mouse thymus. PLoS One (2013) 8:e65196 10.1371/journal.pone.0065196 23750244 PMC3672159

[20] Gu C Yoshida Y Livet J Reimert DV Mann F Merte J Semaphorin 3E and plexin-D1 control vascular pattern independently of neuropilins. Science (2005) 307:265–8 10.1126/science.1105416 15550623

[21] Kim J Oh WJ Gaiano N Yoshida Y Gu C Semaphorin 3E-Plexin-D1 signaling regulates VEGF function in developmental angiogenesis via a feedback mechanism. Genes Dev (2011) 25:1399–411 10.1101/gad.2042011 21724832 PMC3134083

[22] Zygmunt T Gay CM Blondelle J Singh MK Flaherty KM Means PC Semaphorin-PlexinD1 signaling limits angiogenic potential via the VEGF decoy receptor sFlt1. Dev Cell (2011) 21:301–14 10.1016/j.devcel.2011.06.033 21802375 PMC3156278

[23] Shi Q Rafii S Wu MH Wijelath ES Yu C Ishida A Evidence for circulating bone marrow-derived endothelial cells. Blood (1998) 92:362–7 9657732

[24] Purhonen S Palm J Rossi D Kaskenpaa N Rajantie I Yla-Herttuala S Bone marrow-derived circulating endothelial precursors do not contribute to vascular endothelium and are not needed for tumor growth. Proc Natl Acad Sci U S A (2008) 105:6620–5 10.1073/pnas.0710516105 18443294 PMC2365563

[25] Chen Q Khoury M Limmon G Choolani M Chan JK Chen J Human fetal hepatic progenitor cells are distinct from, but closely related to, hematopoietic stem/progenitor cells. Stem Cells (2013) 31:1160–9 10.1002/stem.1359 23404852

[26] Allen JM Forbush KA Perlmutter RM Functional dissection of the lck proximal promoter. Mol Cell Biol (1992) 12:2758–68 1588967 10.1128/mcb.12.6.2758-2768.1992 PMC364470

[27] Cheng J Turksen K Yu QC Schreiber H Teng M Fuchs E Cachexia and graft-vs.-host-disease-type skin changes in keratin promoter-driven TNF alpha transgenic mice. Genes Dev (1992) 6:1444–56 10.1101/gad.6.8.1444 1379563

[28] Fadel BM Boutet SC Quertermous T Functional analysis of the endothelial cell-specific Tie2/Tek promoter identifies unique protein-binding elements. Biochem J (1998) 330(Pt 1):335–43 9461528 10.1042/bj3300335 PMC1219145

[29] Small M Van Ewijk W Gown AM Rouse RV Identification of subpopulations of mouse thymic epithelial cells in culture. Immunology (1989) 68:371–7 2592012 PMC1385450

[30] Surh CD Gao EK Kosaka H Lo D Ahn C Murphy DB Two subsets of epithelial cells in the thymic medulla. J Exp Med (1992) 176:495–505 10.1084/jem.176.2.495 1500857 PMC2119314

[31] Farr AG Anderson SK Epithelial heterogeneity in the murine thymus: fucose-specific lectins bind medullary epithelial cells. J Immunol (1985) 134:2971–7 3856612

[32] Chauvet S Cohen S Yoshida Y Fekrane L Livet J Gayet O Gating of Sema3E/PlexinD1 signaling by neuropilin-1 switches axonal repulsion to attraction during brain development. Neuron (2007) 56:807–22 10.1016/j.neuron.2007.10.019 18054858 PMC2700040

[33] Bleul CC Corbeaux T Reuter A Fisch P Monting JS Boehm T Formation of a functional thymus initiated by a postnatal epithelial progenitor cell. Nature (2006) 441:992–6 10.1038/nature04850 16791198

[34] Surh CD Ernst B Sprent J Growth of epithelial cells in the thymic medulla is under the control of mature T cells. J Exp Med (1992) 176:611–6 10.1084/jem.176.2.611 1500862 PMC2119324

[35] Shores EW Van Ewijk W Singer A Maturation of medullary thymic epithelium requires thymocytes expressing fully assembled CD3-TCR complexes. Int Immunol (1994) 6:1393–402 10.1093/intimm/6.9.1393 7819148

[36] Shinkai Y Rathbun G Lam KP Oltz EM Stewart V Mendelsohn M RAG-2-deficient mice lack mature lymphocytes owing to inability to initiate V(D)J rearrangement. Cell (1992) 68:855–67 10.1016/0092-8674(92)90029-C 1547487

[37] van Ewijk W Shores EW Singer A Crosstalk in the mouse thymus. Immunol Today (1994) 15:214–7 10.1016/0167-5699(94)90246-1 8024681

[38] Levelt CN Mombaerts P Iglesias A Tonegawa S Eichmann K Restoration of early thymocyte differentiation in T-cell receptor beta-chain-deficient mutant mice by transmembrane signaling through CD3 epsilon. Proc Natl Acad Sci U S A (1993) 90:11401–5 10.1073/pnas.90.23.11401 8248261 PMC47990

[39] Fiorini E Schmitz I Marissen WE Osborn SL Touma M Sasada T Peptide-induced negative selection of thymocytes activates transcription of an NF-kappa B inhibitor. Mol Cell (2002) 9:637–48 10.1016/S1097-2765(02)00469-0 11931770

[40] Hirata K Ishida T Penta K Rezaee M Yang E Wohlgemuth J Cloning of an immunoglobulin family adhesion molecule selectively expressed by endothelial cells. J Biol Chem (2001) 276:16223–31 10.1074/jbc.M100630200 11279107

[41] Barcena A Galy AH Punnonen J Muench MO Schols D Roncarolo MG Lymphoid and myeloid differentiation of fetal liver CD34+lineage-cells in human thymic organ culture. J Exp Med (1994) 180:123–32 10.1084/jem.180.1.123 7516402 PMC2191565

[42] Strain AJ Crosby HA Nijjar S Kelly DA Hubscher SG Human liver-derived stem cells. Semin Liver Dis (2003) 23:373–84 10.1055/s-2004-815563 14722814

[43] Baldwin HS Shen HM Yan HC Delisser HM Chung A Mickanin C Platelet endothelial cell adhesion molecule-1 (PECAM-1/CD31): alternatively spliced, functionally distinct isoforms expressed during mammalian cardiovascular development. Development (1994) 120:2539–53 7956830 10.1242/dev.120.9.2539

[44] Zuniga-Pflucker JC T-cell development made simple. Nat Rev Immunol (2004) 4:67–72 10.1038/nri1257 14704769

[45] Halkias J Melichar HJ Taylor KT Ross JO Yen B Cooper SB Opposing chemokine gradients control human thymocyte migration in situ. J Clin Invest (2013) 123:2131–42 10.1172/JCI67175 23585474 PMC3635739

[46] Klug DB Carter C Gimenez-Conti IB Richie ER Cutting edge: thymocyte-independent and thymocyte-dependent phases of epithelial patterning in the fetal thymus. J Immunol (2002) 169:2842–5 12218095 10.4049/jimmunol.169.6.2842

[47] Kurobe H Liu C Ueno T Saito F Ohigashi I Seach N CCR7-dependent cortex-to-medulla migration of positively selected thymocytes is essential for establishing central tolerance. Immunity (2006) 24:165–77 10.1016/j.immuni.2005.12.011 16473829

[48] Davalos-Misslitz AC Rieckenberg J Willenzon S Worbs T Kremmer E Bernhardt G Generalized multi-organ autoimmunity in CCR7-deficient mice. Eur J Immunol (2007) 37:613–22 10.1002/eji.200636656 17304629

[49] Anderson M Anderson SK Farr AG Thymic vasculature: organizer of the medullary epithelial compartment? Int Immunol (2000) 12:1105–10 10.1093/intimm/12.7.1105 10882422

[50] Muller SM Terszowski G Blum C Haller C Anquez V Kuschert S Gene targeting of VEGF-A in thymus epithelium disrupts thymus blood vessel architecture. Proc Natl Acad Sci U S A (2005) 102:10587–92 10.1073/pnas.0502752102 16027358 PMC1180776

[51] Casazza A Finisguerra V Capparuccia L Camperi A Swiercz JM Rizzolio S Sema3E-Plexin D1 signaling drives human cancer cell invasiveness and metastatic spreading in mice. J Clin Invest (2010) 120:2684–98 10.1172/JCI42118 20664171 PMC2912191

[52] Blanc V Nariculam J Munson P Freeman A Klocker H Masters J A role for class 3 semaphorins in prostate cancer. Prostate (2011) 71:649–58 10.1002/pros.21281 20949546

[53] Hughes A Kleine-Albers J Helfrich MH Ralston SH Rogers MJ A class III semaphorin (Sema3e) inhibits mouse osteoblast migration and decreases osteoclast formation in vitro. Calcif Tissue Int (2012) 90:151–62 10.1007/s00223-011-9560-7 22227882 PMC3271215

[54] Movassagh H Shan L Halayko AJ Roth M Tamm M Chakir J Neuronal chemorepellent Semaphorin 3E inhibits human airway smooth muscle cell proliferation and migration. J Allergy Clin Immunol (2013). 10.1016/j.jaci.2013.06.011 23932461

[55] Wanschel A Seibert T Hewing B Ramkhelawon B Ray TD Van Gils JM Neuroimmune guidance cue Semaphorin 3E is expressed in atherosclerotic plaques and regulates macrophage retention. Arterioscler Thromb Vasc Biol (2013) 33:886–93 10.1161/ATVBAHA.112.300941 23430613 PMC3647027

[56] Kemler R Brulet P Schnebelen MT Gaillard J Jacob F Reactivity of monoclonal antibodies against intermediate filament proteins during embryonic development. J Embryol Exp Morphol (1981) 64:45–60 6171607

[57] Klug DB Carter C Crouch E Roop D Conti CJ Richie ER Interdependence of cortical thymic epithelial cell differentiation and T-lineage commitment. Proc Natl Acad Sci U S A (1998) 95:11822–7 10.1073/pnas.95.20.11822 9751749 PMC21724

