# Supplementary figures and images for: Plxnd1 Expression in Thymocytes Regulates Their Intrathymic Migration While That in Thymic Endothelium Impacts Medullary Topology
Source: Front Immunol. 2013 Nov 19;4:392. doi: 10.3389/fimmu.2013.00392 (PMC3832804; doi:10.3389/fimmu.2013.00392)

**A**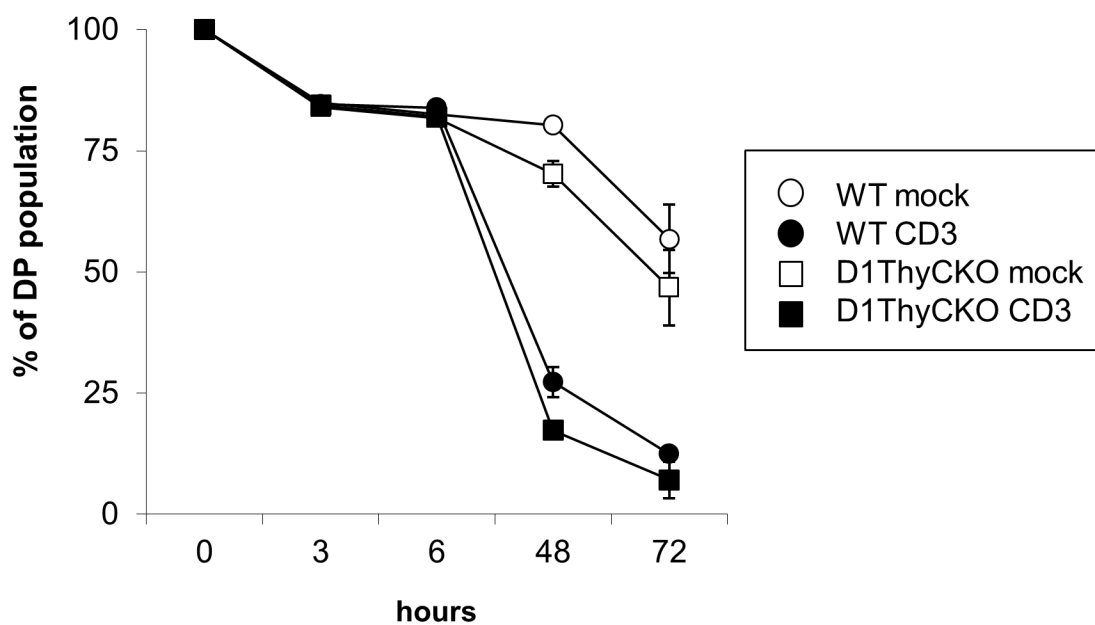**B**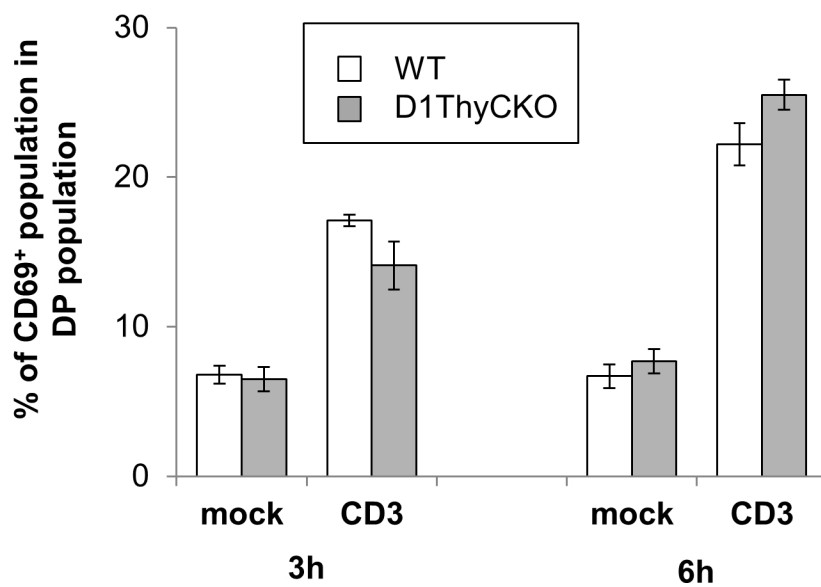

Supplement: Figure S1 — Plxnd1 deletion does not affect apoptosis of, or CD69 expression on, thymocytes following TCR stimulation. (A) One million total thymocytes were stimulated with plate-coated anti-CD3ε mAb for the indicated time period. After incubation, the cells were harvested and stained with anti-CD4-APC/anti-CD8α-PE/anti-TCRβ-FITC mAbs and analyzed by flow cytometry. The %live DP population was normalized against %live DP population at 0 h. Error bars represent SEM. The result is representative of two independent experiments. (B) After stimulation of total thymocytes for the indicated time periods as in (A), the cells were harvested and stained with anti-CD4-APC/anti-CD8α-PE/anti-CD69-FITC mAbs and analyzed by flow cytometry. %CD69+ DP thymocytes was calculated relative to total DP thymocytes. Error bars represent SEM. The result is representative of two independent experiments. [file 71899_Reinherz_Presentation1.PDF]

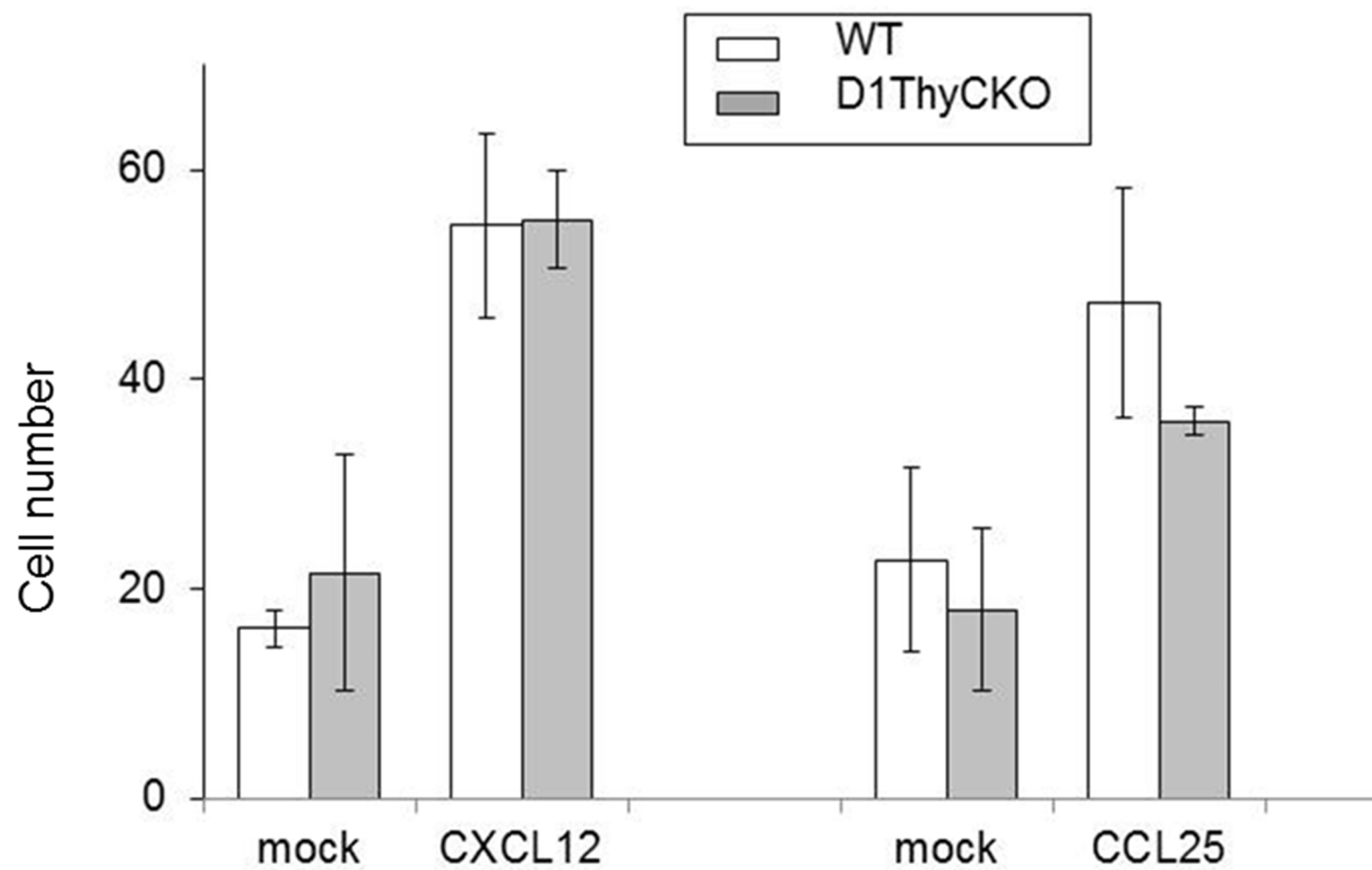

Supplement: Figure S2 — CXCL12- and CCL25-mediated migration is normal in D1ThyCKO mice. Migration assays were performed with CXCL12 and CCL25 as described previously using both chemokines at 1 μg/ml (15). After the incubation for 2 h, the cells migrating across the transwell membrane into the lower well were harvested and counted. White bars depict mock-treated (no chemokine) control experiments. Error bars represent SEM. [file 71899_Reinherz_Presentation2.PDF]

WT

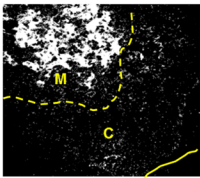

D1ThyCKO

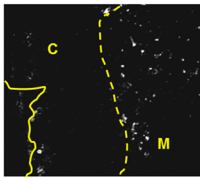

D1EpCKO

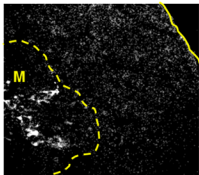

*Sema3e*<sup>-/-</sup>

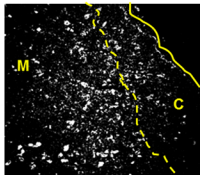

Supplement: Figure S3 — UEA1 expression in plexinD1 conditional knockout and Sema3e−/− thymus. UEA1 expression in sections correlating to those depicted in Figure 2 in the main article. Note that the bulk of UEA1 signal is medullary in all cases. “M” defines medullary area, “C” defines cortical area; the dashed line represents the corticomedullary junction, and the solid line represents the thymic capsule. [file 71899_Reinherz_Presentation3.PDF]

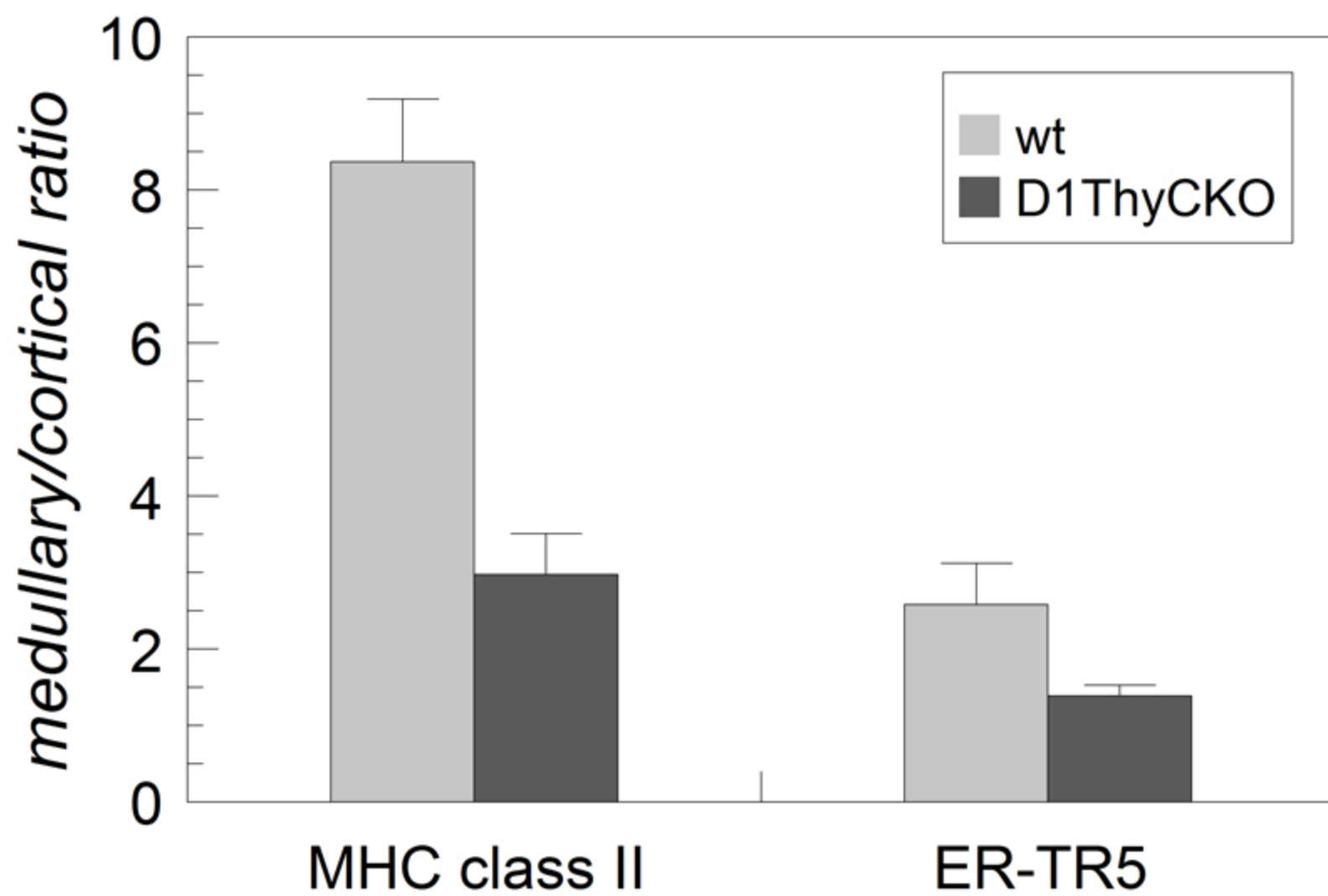

Supplement: Figure S4 — Quantitation of relative cortical and medullary ER-TR5 and MHC class II expression in wt and D1ThyCKO thymi. The relative signals for cortical and medullary-localized fluorescence were determined by image analysis of six D1ThyCKO thymic sections and nine wt sections. For each marker, the color image was converted to a grayscale image, six to seven regions without apparent signal were selected to represent background and the relative intensity for identically sized regions within areas demarcated as cortex or medulla were determined using the ImageJ java suite. The D1ThyCKO medullary MHC class II representation was significantly reduced on comparison with wt (P < 0.005) as was the medullary ER-TR5 representation (P < 0.02). Mean ± SEM presented and compared using Student’s t-test. [file 71899_Reinherz_Presentation4.PDF]

*TCR $\beta$ <sup>+/+</sup> Rag2<sup>-/-</sup>*

24 h

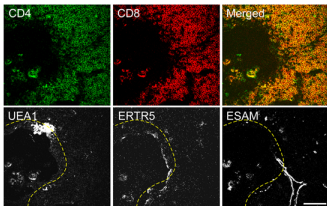

72 h

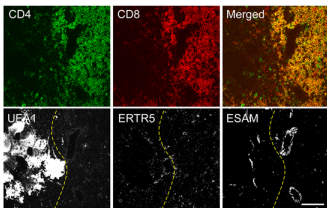

Supplement: Figure S5 — Co-localization of ESAM+ blood vessels and UEA1+ mTEC in thymi of Tcrb+/+Rag2−/− mice injected with anti-CD3ε mAb. Adjacent cryosections of each thymus were stained with the indicated Abs. anti-ESAM was used to visualize endothelial cells and UEA1 to visualize mTEC. The corticomedullary junction is indicated by the dashed line. The result represents two independent analyses. White bar in the ESAM panels is 100 μm and applies to all panels. [file 71899_Reinherz_Presentation5.PDF]

WT

D1ThyCKO

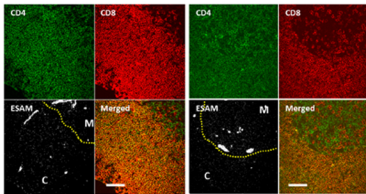*Sema3e*<sup>-/-</sup>*Tcrb*<sup>+/-</sup> *Rag2*<sup>-/-</sup>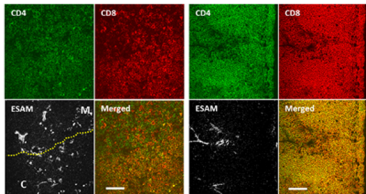

Supplement: Figure S6 — ESAM+ blood vessel distribution is normal in D1ThyCKO mice. The thymic cryosections of the indicated mice were stained as described in Figure 5C. The result is representative of three independent experiments. The dashed line follows the corticomedullary junction where the demarcation between cortex and medulla is based on the location of DP and SP thymocytes, respectively. ESAM is an endothelial cell-specific marker. White bar in merged image is 100 μm. C, cortex; M, medulla. [file 71899_Reinherz_Presentation6.PDF]

# D1EnCKO

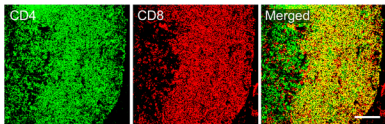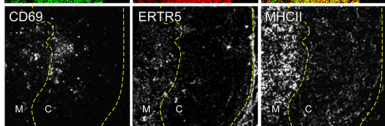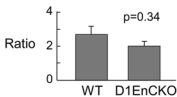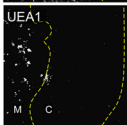

Supplement: Figure S7 — Normal CD69+ thymocyte distribution in transplanted D1EnCKO mice. Adjacent cryosections of D1EnCKO thymi transplanted under the B6.SJL renal kidney capsule were stained as described for Figure 2 and Figure S3. The cortex (C) and the medulla (M) were divided based on the location of DP and SP thymocytes. The left dashed line in each section follows the corticomedullary junction and the right dashed line depicts the thymic capsule. White bar in merged images is 100 μm. The figure is representative of two independent experiments. The graph depicts the enumeration of CD69+ cell density using the FITC signal intensity as described in Figure 3. Sections from three independent thymi were analyzed for each genotype (wt and D1EnCKO) and P values were calculated using Student’s t-test where the ordinate axis depicts the ratio of CD69+ cells in the medulla to CD69+ cells in the cortex. Error bars represent SEM. The P value of 0.34 is not statistically significant. [file 71899_Reinherz_Presentation7.PDF]

**A**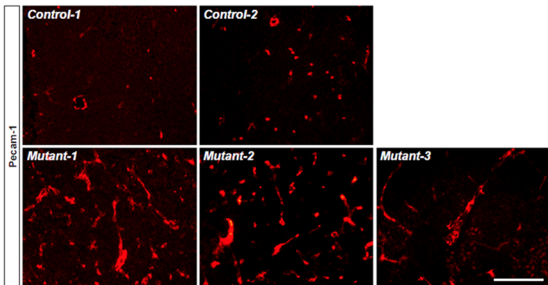**B**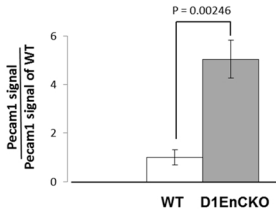

Supplement: Figure S8 — Pecam1+ endothelial cells are increased in newborn D1EnCKO mice. (A) were collected from WT (n = 2, “Control” in figure) and D1EnCKO (n = 3, “Mutant” in figure) newborn mice and fixed overnight in 10% formalin solution, then stained for Pecam1 (CD31). (B) The overall intensity/area was determined by image analysis for WT, and then both WT and D1EnCKO Pecam1 densities were expressed as a ratio of the WT values. Mean ± SEM is depicted. The white bar, applicable to all panels, represents 2 mm. [file 71899_Reinherz_Presentation8.PDF]
